# Vasopressin Bolus Protocol Compared to Desmopressin (DDAVP) for Managing Acute, Postoperative Central Diabetes Insipidus and Hypovolemic Shock

**DOI:** 10.1155/2017/3052102

**Published:** 2017-01-03

**Authors:** Anukrati Shukla, Syeda Alqadri, Ashley Ausmus, Robert Bell, Premkumar Nattanmai, Christopher R. Newey

**Affiliations:** ^1^Department of Neurology, University of Missouri, Columbia, MO, USA; ^2^Department of Pharmacy, University of Missouri, Columbia, MO, USA; ^3^Department of Neurosurgery, University of Missouri, Columbia, MO, USA

## Abstract

*Introduction*. Management of postoperative central diabetes insipidus (DI) can be challenging from changes in volume status and serum sodium levels. We report a case successfully using a dilute vasopressin bolus protocol in managing hypovolemic shock in acute, postoperative, central DI.* Case Report*. Patient presented after bifrontal decompressive craniotomy for severe traumatic brain injury. He developed increased urine output resulting in hypovolemia and hypernatremia. He was resuscitated with intravenous fluids including a dilute vasopressin bolus protocol. This protocol consisted of 1 unit of vasopressin in 1 liter of 0.45% normal saline. This protocol was given in boluses based on the formula: urine output minus one hundred. Initial serum sodium was 148 mmol/L, and one-hour urine output was 1 liter. After 48 hours, he transitioned to 1-desamino-8-D-arginine vasopressin (DDAVP). Pre-DDAVP serum sodium was 149 mmol/L and one-hour urine output 320 cc. Comparing the bolus protocol to the DDAVP protocol, the average sodium was 143.8 ± 3.2 and 149.6 ± 3.2 mmol/L (*p* = 0.0001), average urine output was 433.2 ± 354.4 and 422.3 ± 276.0 cc/hr (*p* = 0.90), and average specific gravity was 1.019 ± 0.009 and 1.016 ± 0.01 (*p* = 0.42), respectively.* Conclusion*. A protocol using dilute vasopressin bolus can be an alternative for managing acute, central DI postoperatively, particularly in setting of hypovolemic shock resulting in a consistent control of serum sodium.

## 1. Introduction

Brain surgery and head trauma are two common causes of central diabetes insipidus (DI) [1–3]. DI can manifest either transiently, permanently, or in a triphasic pattern [1–3]. The three phases of the triphasic pattern are polyuric lasting for 4-5 days, antidiuretic lasting for 5-6 days, and then DI again [1–3]. 1-Desamino-8-D-arginine vasopressin (DDAVP) is a synthetic form of arginine vasopressin and is a primary treatment of central DI [1–3]. Owing to its prolonged duration of action, easy oral/nasal dosing, and beneficial side effect profile, it is the preferred medication in the long-term management of central DI [1–3]. The use of DDAVP, however, in the critically ill patient who develops DI is more challenging. Sodium changes during the triphasic response of central DI in these patients can often be severe [1–3]. Additionally, the critically ill patient may also be fluid underresuscitated and/or hypotensive [4, 5]. Thus, the rationale behind the use of DDAVP in the acute management of critically ill patients with central DI is not clear. 

Ralston and Butt showed that the use of continuous vasopressin infusion in the acute management of DI in the traumatically brain injured patient was associated with a steady decrease in serum sodium, ease of titration, and a huge benefit of physiological adaptation to change in fluid and electrolyte status [6]. Furthermore, the vasopressor effect of vasopressin can be beneficial in the critically ill patient [1, 6]. These physiological homeostatic benefits of vasopressin are advantageous particularly in the critically ill neurological patient. We present a case report comparing two regimens used to manage acute central DI status after neurosurgery in a single patient. The patient was initially treated with dilute vasopressin bolus regimen followed by DDAVP regimen. We compared the efficacy and safety profile of the two regimens by trending the serum sodium, urine output, urine specific gravity, and vitals hourly over the period of institution of the two regimens. 

## 2. Case Presentation

A young, adult male was brought to the emergency department (ED) in a comatose state after he sustained self-inflicted gunshot wound injuries on both his temples and orbits. He was intubated and resuscitated. On arrival, he had a score of 6 on Glasgow coma scale (GCS) and absent pupillary and gag reflexes. Computed tomography (CT) scan showed bifrontal intracerebral contusions, uncal herniation, cerebral edema, and bilateral orbital floor fractures. Emergency bifrontal craniectomy with external ventricular drain (EVD) placement was successfully performed.

Postoperatively, his condition was monitored in the Trauma Unit for four days during the course of which his GCS improved from 6 to 10. Despite the procedure, his intracranial pressure (ICP) recordings continued to peak requiring continued cerebrospinal fluid (CSF) drainage via the EVD. Repeat CT head showed evidence of left parietal extradural hemorrhage. He subsequently had a left parietal craniectomy for clot evacuation. Postoperatively, he was transferred to the neurosciences intensive care unit (NSICU) for further critical care management of his cerebral edema and refractory ICP elevation. On arrival to the NSICU, he was in hypovolemic shock (overall 3.4 L negative fluid balance in past few hours) from clear polyuria. He had tachycardia and hypotension. His sodium was 154 mmol/L. His urine specific gravity was <1.005 and urine osmolality was 123 mOsm/Kg. Given the correlation between his urine osmolality and specific gravity, his DI was subsequently monitored using only urine specific gravity. There was no administration of mannitol or need for corticosteroid replacement.

Given the significant shock state, he was aggressively resuscitated using dilute vasopressin boluses for his DI. This dilute mixture consisted of 1 U of vasopressin in 1 L of 0.45% saline solution. This was given in boluses based on the formula, urine output minus one hundred milliliters.

After starting the dilute vasopressin bolus protocol, the polyuria quickly resolved within 6 hours and sodium decreased. The serum sodium and urine input and output were monitored carefully over the following days. The serum sodium remained in the desired range of 140–145 mmol/L while on vasopressin bolus protocol. Once resuscitated and stabilized, IV DDAVP was started.

Statistical analyses were performed on serum sodium, urine specific gravity, and urine output using student *t*-test with a *p* ≤ 0.05 being considered significant.

**Figure 1:**
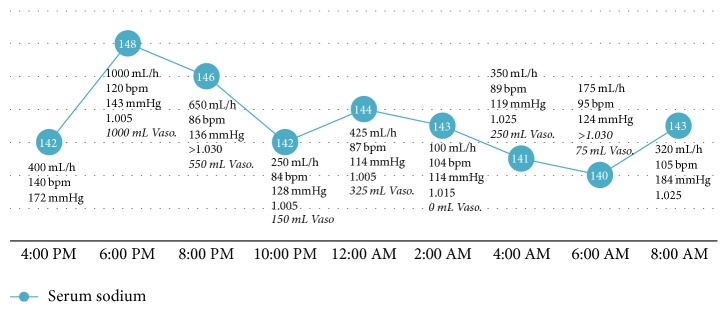
Day 1 vasopressin bolus curve. Sodium (mmol/L; blue circles), urine output (mL/h), heart rate (bpm), systolic blood pressure (mmHg), and urine specific gravity on day 1 of vasopressin/0.45% fluid replacement. Bolus doses of vasopressin replacement are noted to be italic. mL: milliliters; h: hour; bpm: beats per minute; mmHg: millimeters of mercury; vaso: vasopressin.

**Figure 2:**
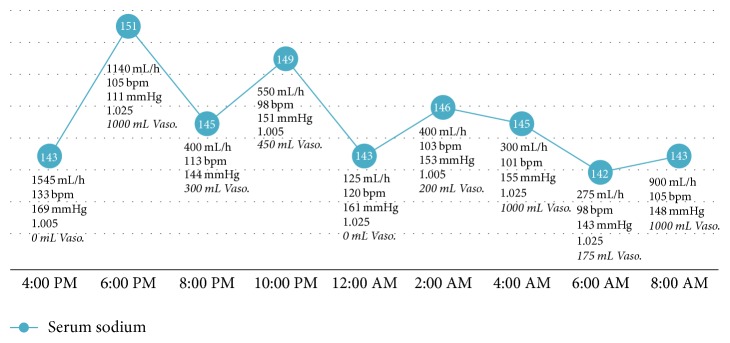
Day 2 vasopressin bolus curve. Sodium (mmol/L; blue circles), urine output (mL/h), heart rate (bpm), systolic blood pressure (mmHg), and urine specific gravity on day 2 of vasopressin/0.45% fluid replacement. Bolus doses of vasopressin replacement are noted to be italic. 1-Desamino-8-D-arginine vasopressin: DDAVP; mL: milliliters; h: hour; bpm: beats per minute; mmHg: millimeters of mercury; vaso: vasopressin.

**Figure 3:**
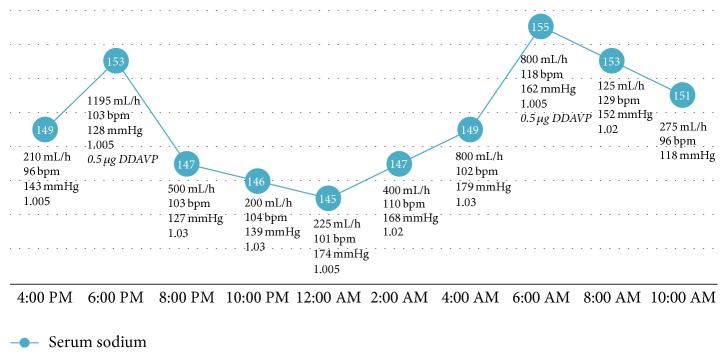
DDAVP curve. Sodium (mmol/L; blue circles), urine output (mL/h), heart rate (bpm), systolic blood pressure (mmHg), and urine specific gravity with DDAVP administrations. DDAVP doses noted to be italic. 1-Desamino-8-D-arginine vasopressin: DDAVP; mL: milliliters; h: hour; bpm: beats per minute; mmHg: millimeters of mercury.

Comparing the bolus protocol to the DDAVP protocol, the average sodium was 143.8 ± 3.2 and 149.6 ± 3.2 mmol/L (*p* = 0.0001), average urine output was 433.2 ± 354.4 and 422.3 ± 276.0 cc/hr (*p* = 0.90), and average specific gravity was 1.019 ± 0.009 and 1.016 ± 0.01 (*p* = 0.42), respectively. The serum sodium, urine output, heart rate, systolic blood pressure, and urine specific gravity (if available) are charted for days 1 and 2 of vasopressin bolus protocol (Figures 1 and 2) and for DDAVP regimen (Figure 3).

## 3. Discussion

Our case shows that the use of a dilute vasopressin urine replacement formula can safely and smoothly treat patients in DI and hypovolemic shock. This protocol provided a consistent serum sodium level compared to the use of DDAVP.

Diabetes insipidus is an endocrinological disorder characterized by polyuria, that is, urine output greater than 2 l/m^2^/24 h or 40–50 mL/Kg/24 h in adults, caused as a result of either vasopressin deficiency (central DI), vasopressin resistance (nephrogenic DI), or excessive water intake (primary polydipsia) [3]. The diagnosis of DI involves first confirming the polyuria (i.e., hypotonic urine) by recording urine output and urine osmolality (or specific gravity) and also the serum sodium [2, 3]. Postoperative DI can typically be managed with drinking to thirst. Water deprivation test is the confirmatory as well as the differentiating test [7]. In patients with normal neurohypophyseal function and patients with primary polydipsia, urine osmolality at the end of period of deprivation was greater than plasma osmolality and failed to increase by more than 5% after 5 units of aqueous vasopressin [7]. In patients with severe ADH deficiency urine osmolality prior to ADH administration was much less than plasma osmolality after it when the urine osmolality was increased by more than 50% [8]. However, in the neurocritical care patient, this thirst mechanism may be impaired or unable to monitor secondary to the critical illness. Management of DI with a defective thirst mechanism demands closer monitoring and observation.

The treatment of DI is targeted towards fluid resuscitation and maintaining normal electrolyte balance status. Hence, volume replenishment is the mainstay of treatment irrespective of the cause. Underlying cause, if identified, must be treated. The primary pharmacological treatment of central DI is with the vasopressin analogue DDAVP. Removal of amine from position 1 of vasopressin increases the half-life of DDAVP and changing 1-arginine to d-arginine at position 8 reduces the vasopressor action [8–10]. DDAVP is available as oral tablets, nasal spray, and a solution [8–10]. For these reasons, it is deemed as a safe and efficacious treatment option for the management of DI [8–10]. In the critical care patient with DI and hypovolemic shock, these features may not be desirable and may result in underresuscitation and/or extreme fluctuation of serum sodium level. The use of dilute vasopressin bolus to replace urine loss may be more desirable.

Vasopressin in continuous infusion has been previously shown to result in a steady decrease in serum sodium with ease of titration resulting in an improvement in fluid and electrolyte status [6]. Furthermore, the vasopressor effect of vasopressin can be beneficial in the critically ill patient who may be in shock as we demonstrate in this case report [1, 6]. Additional studies are needed to assess the efficacy in a larger sample.

## 4. Conclusion

Our case demonstrates that in the acute management of a critically ill patient in DI and hypovolemic shock, dilute vasopressin bolus emerged as a better alternative over DDAVP. Steadier decrease in serum sodium levels, convenience of titration in inpatient setting, and a self-regulating dose modulation in accordance with the body's fluid status conferred a distinct advantage, especially in volume-depleted patients with poor neurological function. Therefore, in critically ill patients, the use of dilute vasopressin should be considered an option.
